# The Neurotoxic Role of Extracellular Tau Protein

**DOI:** 10.3390/ijms19040998

**Published:** 2018-03-27

**Authors:** Álvaro Sebastián-Serrano, Laura de Diego-García, Miguel Díaz-Hernández

**Affiliations:** 1 Department of Biochemistry and Molecular Biology, Veterinary School, Complutense University of Madrid, Avda. Puerta de Hierro S/N, 28040 Madrid, Spain; alvarseb@ucm.es (Á.S.-S.); laura.dediego@ucm.es (L.d.D.-G.); 2 Instituto de Investigación Sanitaria del Hospital Clínico San Carlos, IdISSC, 28040 Madrid, Spain

**Keywords:** tau, TNAP, muscarinic receptors, synaptopathy, Alzheimer’s disease

## Abstract

Tauopathies are a class of neurodegenerative diseases associated with the microtubule-associated protein tau, with Alzheimer’s disease (AD) being the most prevalent related disorder. Neurofibrillary tangles (NFTs) are one of the neuropathological hallmarks present in the brains of AD patients. Because NFTs are aberrant intracellular inclusions formed by hyperphosphorylated tau, it was initially proposed that phosphorylated and/or aggregated intracellular tau protein was causative of neuronal death. However, recent studies suggest a toxic role for non-phosphorylated and non-aggregated tau when it is located in the brain extracellular space. In this work, we will discuss the neurotoxic role of extracellular tau as well its involvement in the spreading of tau pathologies.

## 1. Overview of Tau Protein

Tau protein was initially identified as an intracellular microtubule-associated protein (MAP), involved in the assembly and stabilization of microtubules in a phosphorylation-dependent manner [1,2]. Human tau protein is encoded by the microtubule-associated protein tau gene, *MAPT*, located on chromosome 17q21 that contains 16 exons. Six tau isoforms generated by alternative splicing of exons 2, 3, and 10 have been identified to date [3]. These isoforms may contain 0, 1, or 2 inserts near the amino-terminal end (0N, 1N, or 2N, respectively) and/or the 3 or 4 repeat domains in the carboxy-terminal end (3R or 4R, correspondingly) [4]. The carboxy-terminal repeat domains, which are encoded by exons 9–12, are involved in the interaction between tau and the protein tubulin. The isoforms containing 3R or 4R binding domains result from alternative splicing of exon 10 [5]. The six isoforms of tau have been found in the adult brain; however, only shorter tau is found in the fetal brain. In the adult human brain, the levels of 3R and 4R forms are roughly equal, and their imbalance is sufficient to cause neurodegeneration and dementia [6]. Tau isoforms are mainly found in neurons, although glial cells can also present low levels of them [7]. Subsequent studies revealed that cytosolic tau protein is also involved in other physiological functions, including neural polarization [8], axonal transport [9,10], axonogenesis [11], and regulation of synaptic function and neuronal signaling [12,13]. Besides, although tau protein does not present a nuclear transport signal, its presence in nuclei of both cell lines and human brain cells has been reported [14,15]. At this location, it is postulated that tau protein might play a relevant role in DNA protection [16] and in regulating the cell cycle [17].

Intracellularly, tau protein might undergo different post-translational modifications, such as phosphorylation, glycosylation, isomerization, truncation, glycation, deamidation, nitration, methylation, ubiquitylation, and sumoylation [18]. Changes in tau phosphorylation are known to induce loss of function of the protein by preventing its interaction with microtubules [19]. Native unfolded tau shows a limited tendency to aggregate, although this propensity increases as a result of its phosphorylation [4]. The anomalous accumulation of hyperphosphorylated tau protein leads to the formation of intracellular aberrant aggregates called neurofibrillary tangles (NFTs). These structures are a common histopathological hallmark of a set of neurodegenerative diseases known as tauopathies, including Alzheimer’s disease (AD), progressive supranuclear palsy, corticobasal degeneration, argyrophilic grain disease, Pick disease, Huntington’s disease, and frontotemporal dementia with parkinsonism [20]. On the basis of these findings, it was initially proposed that the phosphorylated and/or aggregated intracellular tau protein was toxic to neurons.

## 2. Extracellular Tau Protein

The development of tauopathies is linked to progressive neuronal loss and cognitive decline [20]. Initial studies found that the number of extracellular tau aggregates (extracellular ghost tangles) was inversely proportional to the number of surviving neurons [21]. Since it was initially postulated that the extracellular tangles came from dead cells which had previously developed intracellular tau aggregates, it was suggested that extracellular tau came from the protein released from dying cells [5]. Supporting this hypothesis, elevated levels of tau were found in cerebrospinal fluid (CSF) samples from AD patients [22]. However, over the last years, a large body of evidence has demonstrated that the source of extracellular tau protein may also be independent of cell death [23]. Thus, the presence of tau has been reported both in the culture media from primary neurons [24] or cell lines overexpressing tau [25] and in the CSF of mice [26] and humans [27]. Other studies have revealed that tau is physiologically released into the extracellular space by neurons in vitro or in mouse brains in vivo. Indeed, these studies have suggested that the release of tau is regulated by neural activity, reporting that stimulation of neuronal activity enhances tau release [23,28]. In this way, an in vivo study using optogenetic and chemogenetic approaches has confirmed that an elevated neuronal activity favors the release of tau and exacerbates tau pathology in the absence of cellular death [29].

## 3. Molecular Mechanisms Proposed for Tau Release

To date, the mechanism underlying tau release remains unknown. Indeed, the lack of a signal peptide on the tau protein sequence that facilitates its translocation to the endosomal reticulum for the conventional secretory pathway has also contributed to the inability to identify this mechanism. Currently, there are four different mechanisms proposed for tau release: direct translocation from the cytoplasm across the plasma membrane, release via secretory lysosomes, microvesicle shedding or exosomes release [4,30]. However, nowadays it still remains unclear which one of these mechanisms is predominant both in physiological and in pathological conditions [4].

A growing body of evidence strongly suggests that tau may be released by presynaptic vesicular secretion [23]. However, tau protein was not found between the protein components of the synaptic vesicles under physiological conditions [31]. It is well known that a specific signal that leads to intracellular tau phosphorylation would favor its dissociation from axonal microtubules, allowing it to reach presynaptic and postsynaptic terminals [2]. In this respect, a study has shown that tau protein may bind to synaptic vesicles via its N-terminal domain, interfering in this way with presynaptic functions, including synaptic vesicle mobility and release rate [32]. Indeed, it was reported that synaptic terminals release tau upon depolarization [29,33].

Interestingly, several groups have reported that tau release may be altered by different external stimuli. For example, it was reported that nutrient deprivation or lysosomal dysfunction lead to an increase of tau secretion [34]. In addition, a high neuronal activity promotes tau release both in cultured neurons and in experimental settings in vivo [23,28,35,36]. Taking into account that neuronal hyperactivity may also regulate tau secretion [36], it has been suggested that an altered tau release may drive to an aberrant cycle of neuronal activation that might contribute to the spreading of the disease [28].

## 4. Clearance of Extracellular Tau

To further understand the role that extracellular tau protein plays both under physiological and under pathological conditions, it is necessary to know how its turnover is regulated at this location, as well as the factors involved. Initial in vitro studies suggested that extracellular tau protein is moderately degraded in the extracellular space, finding that more than 50% of exogenous tau protein added to the culture medium of human neuroblastoma cells remained unaltered the next 48 h [37]. However, later works revealed that the extracellular tau turnover was slower. Using in vivo approaches, it was estimated that soluble tau has a half-life around 9.7 days, whereas its insoluble form may reach a half-life of 34.2 days [38]. This low extracellular clearance rate may underlie the capacity of extracellular tau protein to reach distant regions from its release.

It remains poorly understood what factors are involved in extracellular tau clearance. In vitro studies have suggested that both infiltrated macrophages and microglial cells can phagocytize extracellular oligomerized tau protein, with macrophages being more efficient than microglia in this process. Indeed, microglia cells only appear to be able to degrade the internalized oligomeric tau in a neuroinflammatory environment [39]. Another work has demonstrated that tau is a substrate of both matrix-metalloproteinases MMP-3 and MMP-9, and that a limited proteolysis by MMP-9 leads to an increase in tau oligomer formation [40]. Taking into account the neurotoxic role associated with extracellular tau protein, the recognition of the elements involved in the regulation of extracellular tau would result in the identification of new potential therapeutic targets to treat tauopathies.

## 5. Extracellular Tau Contributes to Synaptic Dysfunction in AD

Another of the major hallmarks of the AD is the extensive loss of synapses [41] that correlates with cognitive impairment [42]. Although initial studies postulated that amyloid-beta induced the synaptotoxicity observed in AD, in the last years, accumulated evidence indicates that tau plays a key role on synaptic reduction. Thus, a recent work has suggested that phosphorylation of tau protein is required for amyloid-beta-induced synapse loss [43]. Furthermore, a strong association between the presence of tangles with hyperphosphorylated tau and a reduction in presynaptic protein expression was found in the brain of AD patients [44]. Hence, the expression of the synaptic vesicle-associated protein synaptophysin is reduced in tangle-containing neurons compared with adjacent tangle-free neurons [45]. Another study has demonstrated that depolarization-induced tau release is significantly increased in AD patients, postulating that this abnormal tau-secretion from the presynaptic compartment may be contributing to the synaptic dysfunction associated with AD [46]. Besides, it was postulated that the blocking of synaptic transmission induced by the microinjection of human tau into the presynaptic terminal of the squid axon could be due to an alteration of the docking of synaptic vesicles [47]. Furthermore, since synaptic decline has been detected at early stages of AD, neuronal loss is not enough to explain synapse loss, suggesting that synaptic pruning precedes neuronal death [48,49]. In agreement with the relevance of tau in the synaptic dysfunction related to AD, it was reported that the addition of extracellular tau oligomers impairs memory long-term potentiation in mice [35,50]. Besides, another work showed that the application of low-n-oligomers of tau to neurons in culture affects the morphology and density of dendritic spines. Interestingly, this effect was accompanied by increased expression of reactive oxygen species and alterations in intracellular calcium homeostasis [51].

In another interesting work, different inducible transgenic mice expressing full-length or truncated tau were generated to elucidate the influence of both tau species in the synaptic dysfunction related to tauopathies [52]. The results revealed that both tau variants were able to induce AD features, including synapse loss. However, while full-length tau led to a “pre-tangle” pathology, truncate tau caused a more severe pathology, including the massive formation of NFTs and hippocampal neuronal loss [52]. Since the authors of this study used conditional mice models, they also reported that memory and synapses impairments were recovered after switching off the expression of tau, demonstrating in this way that tauopathies may be reversible once pathological tau is removed. The relevance of truncated tau in AD pathology was also revealed by the observation that the majority of cortical synaptic terminals isolated from AD patients presented a tau protein lacking the C-terminus. Indeed, only 15% of the synaptic terminals isolated from AD patients contained full-length tau [46]. Several works have reported that tau fragments containing the N-terminal domain of human tau can interact with the plasma membrane [53], inducing synaptotoxicity [54]. Intriguingly, the most significant part of tau protein secreted from neurons independently of cell damage is the C-terminally truncated form [55]. Indeed, another study revealed that in AD patients the potassium-induced release of tau fragments from presynaptic terminals was increased compared to that detected in healthy subjects [46]. On the other hand, another study using a synthesized peptide containing the tau N-terminal domain, revealed that soluble tau could contribute early to the pathology at the extracellular level, causing synaptic alterations independently of overt neurodegeneration [56]. At this point, it is relevant to remind that, although full-length tau has been detected in the CSF from healthy humans [57], fragments of this protein, including the N-terminal domain, have been only identified in CSF samples from AD subjects [58,59]. Nonetheless, it is still debated which tau species, the full-length or the truncated form, has the major impact on synaptic dysfunction related to tau pathology.

## 6. Extracellular Tau Induces a Neurotoxic Effect via Muscarinic Receptor. Involvement of Tissue-Nonspecific Alkaline Phosphatase

Together with senile plaques and NFTs, another major hallmark of AD is a basal forebrain cholinergic hypofunction. The degeneration of cholinergic neurons is considered an early pathological event together with tangle formation [60] and it is also related to the cognitive impairment observed in AD [61]. In brain from AD patients, loss of cholinergic fibers and nerve terminals and reduction of cholinergic receptors were detected [60]. Interestingly, the deficit of muscarinic receptors observed in AD has been related to the impaired neurotransmission associated with the disease [62], suggesting that cholinergic depletion may be the underlying mechanism of synaptic dysfunction. Supporting this hypothesis, muscarinic agonist drugs have shown to have therapeutic value to delay the progression of the AD, improving cognitive behavior and also reducing tau phosphorylation and amyloid-beta aggregation [63,64,65].

One of the first works describing the extracellular tau-induced neurotoxic effect was carried out by Gomez-Ramos et al. using an in vitro approach [66]. In this work, the authors reported that exogenous tau addition could induce a toxic effect on cultured hippocampal neurons. The identified molecular mechanism underlying this neurotoxic effect was a sustained increase of intracellular calcium induced by extracellular tau that caused an unbalance in cellular calcium homeostasis leading to cell death [64]. Similar mechanisms were found in other neurodegenerative disorders [67,68]. Furthermore, the authors suggested that soluble tau (composed mostly of monomers and small oligomers) but not purified large tau aggregates (paired helical filaments, PHFs) are the neurotoxic species [66].

In a later work, muscarinic receptors M1 and M3 were identified as the plasma membrane receptors involved in the neurotoxic effect induced by tau [69]. In agreement with these findings, a muscarinic receptor binding region was found in the C-terminal domain of tau protein, including residues 391–407 [69]. It should be noted that M1/M3 receptors have also been related to the amyloid processing of amyloid precursor protein (APP), therefore alterations in this signaling could also have consequences in the generation of senile plaques leading to a higher severity of AD pathology [70].

Once the muscarinic receptors were identified as the extracellular tau target receptors, the question that arose was why tau, and not acetylcholine (ACh), is able to induce a neurotoxic effect through muscarinic receptor activation. To address this question, the kinetic parameters of muscarinic receptors activated by ACh or tau protein were analyzed. These studies revealed that the affinity of the muscarinic M1 and M3 receptors for tau protein is 10 times higher than that for ACh. Moreover, ACh induced muscarinic receptor desensitization, but tau did not [37]. Interestingly, it was also reported that repetitive stimulation of neuronal muscarinic receptor by tau might cause a sustained increase of intracellular calcium levels that result in cell death [37].

Subsequent works studying the neurotoxic effect induced by extracellular tau described that this protein has to be in its dephosphorylated form to act as a muscarinic receptor agonist. In this respect, tissue-nonspecific alkaline phosphatase (TNAP) was identified as one of the extracellular enzymes involved in tau dephosphorylation [71]. Interestingly, the specific activation of muscarinic receptors by dephosphorylated tau induces TNAP expression and phosphorylation of intracellular tau [71]. Besides, in this work, the authors described that brain samples from Alzheimer’s disease patients presented increased protein and messenger levels of TNAP as well as a higher TNAP enzymatic activity than samples from healthy controls [71]. In good agreement with these observations, a preclinical assay with more than 100 AD patients also revealed that TNAP activity was significantly increased both in the hippocampus and in the blood of the AD patients, independently of sporadic or familial AD diagnosis [72]. It should be noted that TNAP has also been related to the regulation of axonal growth and branching. In fact, it was reported that during the neurogenesis, when neurons acquire their specific morphology and a neurite develops into axon, a significant increase of TNAP expression in the neuronal axonal growth cone occurs. Functional studies confirmed that both pharmacological inhibition and specific knockdown of TNAP reduced axonal growth and branching [73]. Indeed, it was demonstrated that transgenic mice lacking TNAP suffer neurodevelopmental alterations due to dysfunctions affecting axonal elongation and the dendritic tree of hippocampal and cortical neurons [74]. Therefore, the increased expression detected in the brain of AD patients may be part of the physiological response to counter the synaptic loss associated with the disease. Moreover, at its presynaptic location in the central nervous system (CNS), TNAP is involved in the metabolism of several compounds relevant for synaptic functionality. For instance, TNAP regulates the intracellular concentration of pyridoxal 5′-phosphate (PLP) [75], a cofactor of GAD65 [76] essential for gamma-aminobutyric acid (GABA) synthesis. Since this neurotransmitter promotes the specific phosphorylation of tau at the AT8 epitope via GABA_A_ receptor in cultures of mature cortical neurons [77], the increased expression of TNAP may lead to the phosphorylation of intracellular tau, promoting synaptotoxicity. On the other hand, TNAP also regulates the extracellular hydrolysis of nucleotides [78], controlling, in this way, the signaling mediated by these compounds. Considering that presynaptic nucleotide receptors are involved in the vesicular release of numerous neurotransmitters, such as ACh, Glutamate, or GABA [79,80,81], and also favor amyloid processing of APP [82], the alteration of nucleotide signaling by changes of TNAP expression may contribute both to the synaptopathy and to the generation of senile plaques detected in AD.

## 7. Spreading of Tau Pathology

Over the last years, growing evidence has been accumulated suggesting that the spreading of pathological tau occurs predominantly through neuron-to-neuron transmission [4]. This hypothesis is supported by different studies showing that intracranial injection of various forms of tau protein, such as fibrils [83,84], filaments [85], oligomers [86], or monomers [87], have seeding properties, inducing tau pathology spreading from the injection site to anatomically connected brain areas. Although these works suggest that neuronal connectivity could be playing a key role in neuronal vulnerability as well in the spreading of tau pathology [88], the specific mechanism involved remains unknown. Interestingly, in a study, it was reported that in vivo injection of synthetic tau fibrils can induce selective neuronal loss in the surrounding area together with tau pathology spreading to connected brain areas [83]. Additional studies have postulated that monomeric tau protein is the minimal initiating element able to induce tau pathology spreading [87]. Nevertheless, another work suggests that both tau oligomers and monomers foster tau pathology albeit through different mechanisms. According to this study, tau oligomers would be involved in tau spreading, favoring its assembly through a nucleation mechanism, whereas tau monomers would be inducing neurodegeneration and apoptotic cell death [89].

**Figure 1:**
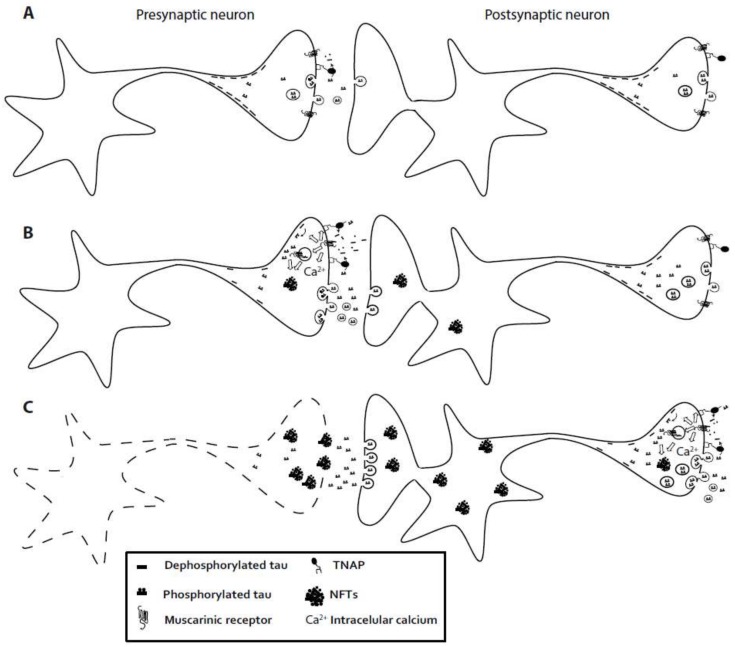
Schematic representation illustrating the involvement of TNAP in the progression of Alzheimer’s disease. (**A**) Under physiological conditions, a moderate amount of tau protein can reach the interstitial space by different mechanisms. (**B**) As a consequence of an initial undetermined event, an unusual neuronal activity is induced, increasing tau secretion into the extracellular space. At this location, tau is dephosphorylated by TNAP becoming an active ligand of muscarinic receptors. The activation of muscarinic receptors by tau has four main consequences: First, a sustained increase in intracellular calcium levels that could compromise intracellular calcium homeostasis; Second, an increase of intracellular tau phosphorylation favoring the formation of NFTs; Third, higher expression levels of TNAP; Finally, possible receptor internalization following tau-induced muscarinic receptor activation. As result of these events, tau secretion would be increased, generating a positive feedback loop at the presynaptic level that finally leads to synaptic dysfunction. In the meantime, at the postsynaptic level, internalization of extracellular tau protein would take place. (**C**) As a result of this vicious cycle, neuronal functionality could be compromised, causing the rupture of the plasma membrane of the presynaptic neuron, with release of its intracellular content, including the NFTs, into the interstitial space. Since NFTs will undergo a slow disassembly and degradation, they will behave as new sources of extracellular tau, promoting the postsynaptic uptake of extracellular tau and the generation of NFTs at postsynaptic level. Finally, this event will stimulate the phosphorylation of intracellular tau resulting in a higher tau secretion also in the postsynaptic neuron, thus favoring the vicious cycle described above and the spreading of the disease.

Taking into account all the findings described above, we could postulate the following theory about the pathological spreading of tau (Figure 1). As a consequence of an initial unknown event that increases the extracellular levels of tau and its subsequent dephosphorylation by TNAP, an unusual activation of muscarinic receptors occurs in the affected area. This uncommon activation promotes the phosphorylation of intracellular tau and the increased expression of TNAP. The phosphorylation of intracellular tau would prevent its interaction with microtubules, favoring its localization at the synaptic terminals which would lead to an unusual tau release, eventually causing synaptic dysfunction. Besides, it has been reported that muscarinic M1 receptors may undergo agonist-induced receptor internalization (or sequestration) [90], so their activation by the extracellular dephosphorylated tau protein could mediate the internalization of extracellular tau. In addition, both pre and post-synaptic neurons may internalize or take up extracellular tau protein, by a still unknown mechanism, which would also favor intracellular tau nucleation and generation of NFTs. As a consequence of both events, NFTs formation will be exacerbated in the neurons of this region, increasing tau release and thus favoring the spreading of tau and the related synaptic dysfunction. Further, the increased expression of TNAP induced by extracellular tau would produce a more efficient dephosphorylation of tau itself, closing a positive feedback loop. This scenario will compromise cell viability by disturbing cellular homeostasis, first affecting the cells closest to the focus of initial tau release. Subsequently, the rupture of the plasma membrane would lead to the release of intracellular content into the interstitial space where the NFTs will provide a new source of extracellular tau. Moreover, due to the low disassemble rate of NFTs, the associated tau release will be sustained on time, contributing to the spreading of this aberrant cycle of stimulation, cell death, and tau release to new surrounding regions [78].

## 8. New Therapeutic Strategies Targeting Extracellular Tau

On the basis of the relevant role that extracellular tau protein has both in the spreading and in the pathology associated to tauopathies, a new set of promising therapeutic approaches are currently being evaluating. The first one is focused in immunotherapy. Thus, administration of anti-tau protein antibodies has demonstrated an improvement of the pathology in a transgenic mice model of AD [91]. Moreover, antibodies that recognize and remove extracellular tau have been successfully used in mouse models [92]. However, despite these promising results, it is debated whether tau antibodies limit their action extracellularly or if they can eventually enter the cells. In this regard, neuronal uptake of antibodies via clathrin-dependent Fcγ receptor endocytosis has been reported [93]. Also, it is discussed which, between active and passive immunization, could be the most effective therapeutic approach. Nevertheless, taking into account that the administration of extracellular tau may trigger the pathology and that passive immunization is not permanent and therefore less practical, additional therapeutic strategies should be considered [94].

Post-translational modifications of tau may influence its half-life in the extracellular location, e.g., soluble tau phosphorylated in the proline-rich region is cleared faster than unphosphorylated soluble tau extracellularly [38]; therefore, the identification of factors capable to modify tau protein in the extracellular location could open new avenues for other therapeutic interventions. In this regard, considering the ability of TNAP to dephosphorylate extracellular tau, it was postulated that specific inhibitors of this ectoenzyme might have therapeutic effects, favoring the clearance of extracellular tau and also avoiding the neurotoxic effect related to tau-induced aberrant activation of the muscarinic receptors [71]. However, additional studies need to be done in order to validate this hypothesis.

Taking into account that tau knockout mice do not show overt pathology [95] and that a decrease of tau protein mitigates amyloid-dependent toxicity, it was postulated that the reduction of tau expression may be a putative therapeutic strategy. In vivo studies using antisense oligonucleotides that selectively reduce endogenous tau expression in the CNS have been shown to efficiently reduce neuronal hyperexcitability in adult mice [96]. However, additional studies should be done to confirm the efficiency of this therapeutic strategy for the treatment of the disorders characterized by tau-mediated neuronal hyperexcitability. Using a similar reasoning and keeping in mind the existence of extracellular elements capable to degrade extracellular tau promoting its clearance, another new therapeutic strategy would be to promote the enzymatic activity of these elements to favor extracellular tau removing.

## 9. Conclusions

In summary, the studies presented here demonstrate that, in addition to its well-established intracellular functions, in the CNS tau protein may be released into the extracellular space via different mechanisms in both physiological and pathological conditions. However, it remains unknown which location is the predominant in each condition. Extracellular tau protein, which presents a low clearance rate at this location, has been involved in different physiological and pathological events. The presence of tau protein in the synapses of healthy brains suggests tau involvement in synaptic functionality. However, the significant loss of synapses detected in tauopathies has indicated that this phenomenon may be partly due to increased toxicity of tau at this location. Although the molecular mechanism involved in extracellular tau-mediated neurotoxic effect still remains unknown, different studies have suggested that the neurotoxic form of tau is soluble rather than aggregated as the one present in tangles. This hypothesis could explain the failure of the therapeutic approaches based on the use of inhibitors of tau aggregation. In fact, it has been reported that a tau aggregation blocker failed to induce benefits in phase III trials [97]. Thanks to the recent advances made that help us to understand that extracellular tau plays a critical role in tauopathies, new therapeutic strategies are currently being explored to treat these disorders. These new approaches are mainly focusing on limiting, blocking, or removing tau protein from the extracellular space. For this reason, the identification and characterization of the elements promoting or involved in extracellular tau degradation, would favor the development of novel and promising therapeutic strategies for the treatment of tauopathies.

## Abbreviations

AD	Alzheimer’s disease
NFTs	Neurofibrillary tangles
CSF	cerebrospinal fluid
MMP	matrix-metalloproteinases
PHFs	paired helical filaments
APP	amyloid precursor protein
ACh	acetylcholine
TNAP	tissue-nonspecific alkaline phosphatase
PLP	pyridoxal 5′-phosphate
GABA	gamma-aminobutyric acid
CNS	central nervous system
